# Brain Iron Homeostasis and Mental Disorders

**DOI:** 10.3390/antiox12111997

**Published:** 2023-11-13

**Authors:** Qiong Wu, Qiuyang Ren, Jingsi Meng, Wei-Juan Gao, Yan-Zhong Chang

**Affiliations:** 1 Hebei Key Laboratory of Chinese Medicine Research on Cardio-Cerebrovascular Disease, Hebei University of Chinese Medicine, Shijiazhuang 050200, China; wuqiong@hebcm.edu.cn; 2 Ministry of Education Key Laboratory of Molecular and Cellular Biology, The Key Laboratory of Animal Physiology, Biochemistry and Molecular Biology of Hebei Province, College of Life Sciences, Hebei Normal University, No. 20 Nan’erhuan Eastern Road, Shijiazhuang 050024, China; renqiuyang1026@163.com (Q.R.); mengjingsi1208@163.com (J.M.)

**Keywords:** brain iron, mental disorders, oxidative stress, neurotransmission, myelination

## Abstract

Iron plays an essential role in various physiological processes. A disruption in iron homeostasis can lead to severe consequences, including impaired neurodevelopment, neurodegenerative disorders, stroke, and cancer. Interestingly, the link between mental health disorders and iron homeostasis has not received significant attention. Therefore, our understanding of iron metabolism in the context of psychological diseases is incomplete. In this review, we aim to discuss the pathologies and potential mechanisms that relate to iron homeostasis in associated mental disorders. We propose the hypothesis that maintaining brain iron homeostasis can support neuronal physiological functions by impacting key enzymatic activities during neurotransmission, redox balance, and myelination. In conclusion, our review highlights the importance of investigating the relationship between trace element nutrition and the pathological process of mental disorders, focusing on iron. This nutritional perspective can offer valuable insights for the clinical treatment of mental disorders.

## 1. Introduction

Mental disorders, also referred to as mental health conditions, are afflictions that impact cognition, emotion, and behavioral regulation, significantly impeding both children’s learning abilities and adults’ functionality in their families, workplaces, and society at large [1]. These disorders typically manifest early in life and often exhibit a chronic recurring pattern. Due to their high occurrence, early onset, persistent nature, and associated impairments, mental disorders contribute significantly to the overall disease burden. While disability constitutes the primary burden stemming from these disorders, premature mortality, particularly resulting from suicide, should not be underestimated [2]. The etiology of mental disorders is multifaceted. A vast corpus of research has linked the origins of such disorders intimately to aspects of neuronal function, myelination, and neurotransmission. What began as a focus on abnormalities within distinct brain areas in early research has evolved into a comprehensive exploration of the deficiencies in neural systems and synaptic plasticity within neural regions relevant to mental health [3,4,5,6]. Over the past decade, a body of evidence has emerged suggesting that mental disorders are often accompanied by dysfunctional myelination changes, myelin sheath defects, and correlated alterations in white matter. This underlying pathology potentially compromises inter-neuronal information transfer, processing, and communication, which can feasibly culminate in behavioral, sensory–motor, affective, and cognitive symptoms manifesting in patients with mental disorders [7,8,9,10]. Numerous studies have additionally highlighted alterations in dopamine, serotonin, and glutamatergic neurotransmission in mental disorders, including, but not limited to, depression, cognitive disorders, and schizophrenia [11,12,13].

As a critical component in several proteins, iron is involved in numerous processes, such as oxygen transport, oxidative phosphorylation, myelin production, and the synthesis and metabolism of neurotransmitters [14]. We and others have demonstrated that altered iron metabolism leads to deleterious imbalances of homeostatic processes throughout the body. Iron overload will aggravate oxidative stress through Fenton chemistry, which can induce cell death and is related to several pathological processes, such as several neurodegenerative diseases [15,16,17]. Conversely, inadequate iron, either peripherally or centrally, also has profound effects on health. Iron deficiency is the most widespread single nutrient deficiency worldwide [18], and can lead to decreased immune function, stunted growth and impaired thermoregulation [19]. Iron deficiency anemia (IDA) in adolescents can even result in abnormal development of the central nervous system (CNS) [20,21].

At the cellular level, iron also plays an essential role as a cofactor for various enzymes in biological systems. Its unique properties, such as its ability to obtain and lose electrons, make it invaluable in many vital biochemical processes. The key role of iron as an enzyme cofactor is pivotal to many biological processes, ranging from energy production and oxygen transport to DNA synthesis and repair [22,23]. Additionally, this metal participates in the synthesis of various neurotransmitters including monoamines, functioning as a cofactor of aromatic amino acid hydroxylases (phenylalanine, tyrosine, tryptophan [Trp]), which is the limiting step in the synthesis of monoamines, whose levels can affect many mental disorders, including depression [24]. Iron is also essential for normal myelin production and maintenance. Dysregulated myelination can lead to psychiatric diseases, including schizophrenia, depression, and autism [25]. Thus, it is likely that iron plays an important role in the occurrence and processes surrounding mental disorders. In this review, we focus on the relationship between brain iron homeostasis and the pathogenesis of mental disorders, with an eye toward the potential molecular mechanisms connecting this essential metal to brain function.

## 2. Regulation of Brain Iron Homeostasis

### 2.1. Iron Uptake into the Brain

Outside the brain, the vast majority of circulating iron is derived from macrophages, with small, but essential, contributions from enterocytes and hepatocytes. The blood–brain barrier (BBB), a physical and metabolic barrier between the CNS and the blood circulation system [26,27], is the major route of iron entry into the brain from the periphery [27,28]. The BBB is composed of brain microvascular endothelial cells (BMVECs), including tight junctions, astrocytes (end-feet) and pericytes, all of which not only form the neurovascular unit that affords structural and inductive support, but also act as a controllable and selectable transport and metabolic system, playing an important role in protecting the brain from harmful, polar molecules, maintaining the physiological function of neurons [29,30,31].

The endothelial cells of BBB control iron uptake as an “entrance guard” [32,33]. Mathematical modeling has suggested that the endothelial cells can regulate both transferrin-bound iron (Tf-Fe) and the non-transferrin-bound iron (NTBI) entry into the CNS by controlling both the expression and internalization of transferrin receptor 1 (TfR1), and acidification of endocytic vesicles [34,35]. Overall, iron transport across the BBB involves two transmembrane steps: iron uptake into BMVECs at the luminal membrane (on the blood side), followed by iron efflux into the brain interstitium at the abluminal membrane (on the brain side) [27]. Various studies suggest that the Tf-TfR1 pathway is the primary route for iron transport across the luminal membrane of BMVECs [36,37,38]. To cross the abluminal membrane, iron export is mediated by ferroportin1/hephaestin (FPN1/HP) and/or FPN1/ceruloplasmin (CP). After iron efflux from the brain side of BMVECs through FPN1, the ferrous iron (Fe^2+^) transported by FPN1 is oxidized to ferric iron (Fe^3+^) by CP or HP. The Fe^3+^ can then be incorporated into apo-Tf circulating in the brain for iron uptake to neurons or glial cells [39,40]. Other pathways may also be involved in iron transport across the BBB, such as lactoferrin receptor/lactoferrin and Glycosylphosphatidylinositol-anchored (GPI-anchored) melanotransferrin/soluble melanotransferrin [41,42]. In addition to these pathways, Tf-Fe may also be transferred across the BBB through transcytosis [43,44]. However, the comprehensive regulatory mechanisms of iron entry across the BBB in vivo still require further elucidation. Cerebrospinal fluid can also be the iron entry route for the brain across the choroid plexus [45]. It is speculated that iron trafficking into the cerebrospinal fluid is similar to the route of iron across the BBB [37,46].

### 2.2. Iron Export from the Brain

After iron enters the brain, it is then released into the brain interstitial fluid. Brain cells, including neurons, astrocytes, oligodendrocytes and microglia, may take up Tf-Fe or NTBI via TfR1/divalent metal transporter 1 (DMT1) or DMT1 pathways, respectively; due to the presence of TfR1 and DMT1 on the membranes of these cells, they may also have additional, specific mechanisms of iron uptake [47]. It must be noted that different brain regions contain diverse iron contents [48,49], with relatively abundant levels in the basal ganglia region, subtantia nigra (SN), red nucleus and cerebellar dentate gyrus [50,51]. In contrast, iron is relatively low in the cerebral cortex, white matter and medulla oblongata [52,53].

Imported iron is usually used for neuronal and glial metabolism, with excess iron either stored in ferritin or released from the brain. Importantly, FPN1, also referred to as Slc40a1, MTP1, or Ireg1, is the sole protein responsible for cellular iron release in mammals [54]. FPN1 is widely expressed in brain cells, including those in the choroid plexus, ependyma, and the BBB [26,55]. Thus, it is not surprising that FPN1 plays a pivotal role in regulating brain iron homeostasis through mediating iron efflux from the BBB. We have reported that FPN1 ablation in neurons and astrocytes leads to iron deficiency in the cortex and hippocampus [26]. In addition, disrupted expression of FPN1 in BMVECs also shed light on the mechanism of iron delivery to the brain through the BBB [56]. Finally, we found that the formation of fear memory was impeded after neuronal FPN1 depletion [26]. Our results provide clues into the role played by brain iron in mental disorders, possibly identifying potential targets for the treatment of post-traumatic stress disorder (PTSD), which is characterized by abnormally persistent and distressing fear memory [26,57].

### 2.3. Regulation of Iron in the Brain

At the cellular level, the regulation of cellular iron levels is primarily controlled at the translational level by iron-responsive element (IRE)-binding proteins (IRP1 and IRP2), despite some control existing at the transcriptional level as well [58,59]. In the context of mental health, IRP2, particularly through its regulation of ferritin expression, has been significantly associated with schizophrenia. It has been observed that IRP2 is marginally overexpressed in the prefrontal cortex of individuals with schizophrenia, leading to lower levels of ferritin expression among patients with this condition [60]. Furthermore, mutations in other genes involved in controlling brain iron homeostasis, such as ferritin and CP, can also contribute to disorders of brain iron metabolism and impact myelin synthesis [61,62]. At the systemic level, hepcidin, a key regulator of iron, plays a vital role in modulating iron metabolism. Hepcidin combines with and subsequently degrades FPN1 to limit extracellular iron availability. Within the brain, the cells responsible for iron regulation (possibly astrocytes) sense the levels of extracellular iron and adjust hepcidin secretion accordingly to ensure appropriate iron transport at the BBB [56,63].

**Figure 1:**
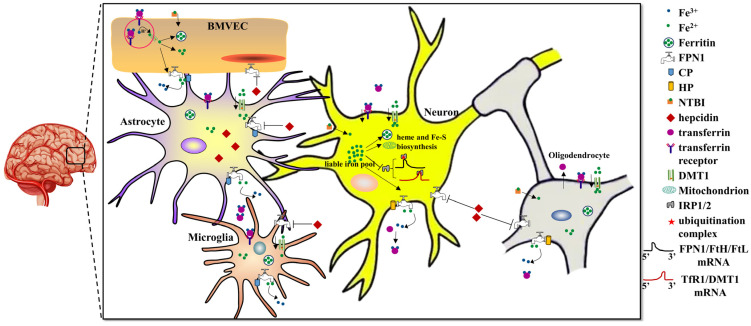
Systemic and cellular regulation of brain iron metabolism.

In summary, the BBB displays an important role in controlling brain iron levels because of its specialized anatomical and physiological functions in iron uptake from the circulatory system (Figure 1). Importantly, the brain needs a comparatively stricter regulation of iron metabolism for optimal cell functioning. Dysregulation of brain iron can lead to either local iron accumulation, resulting in neurodegenerative diseases, iron deficiency, or giving rise to brain development problems. Both iron accumulation and iron deficiency may cause psychiatric disorders [24]. Hence, it is crucial to comprehensively understand the mechanisms of iron transport across the BBB to evaluate and eventually affect brain iron in the field of mental health.

Brain iron uptake mainly occurs through the BBB via Tf-TfR1/DMT1 in the apical surface of BMVECs. FPN1 is transporter responsible for iron export from the BMVECs with the help of CP expressed from astrocytes. After iron influx into the brain, other brain cells, including astrocytes, neurons, oligodendrocytes and microglia, may, respectively, take up Tf-Fe or NTBI via the TfR1/DMT1 or DMT1 pathways. Iron in brain cells is used for electron transport biosynthesis of hemoproteins, Fe-S proteins and neurotransmitters. Excess iron will be either stored in ferritin or exported by FPN1 with oxidation of the extracellular metal by CP or HP. The other ferrous iron can be found in a soluble, chelatable state, which constitutes the labile iron pool. Hepcidin plays a key role in regulating brain iron homeostasis. Hepcidin that is mainly synthesized from astrocytes can sense extracellular iron levels and then bind to FPN1 and promote its degradation, ultimately decreasing cellular iron release. Cellular iron metabolism is primarily regulated by the IRE/IRP system. In iron deficient cells, IRPs bind to the 5′-IREs in ferritin and FPN1 mRNAs to repress transcription. In contrast, TFR1 and some DMT1 variants contain 3′ UTR IREs, which bind IRP2 under iron deficiency, stabilizing the mRNA, which guarantees the synthesis of iron importers.

## 3. Iron and Mental Disorders

Numerous mental disorders have been described, with signs and symptoms that vary widely between specific disorders [64]. Common disorders include depression/anxiety disorders, schizophrenia, fear disorders, including PTSD, and neurodevelopment disorders, including ADHD and autism spectrum disorder (ASD), which usually occurs early in development [65]. The stigma and discrimination associated with mental disorders can add to the suffering and disability, leading to social movements to increase awareness and challenge social exclusion [66]. In this section, we mainly discuss the relationship between brain iron metabolism and the pathology of mental disorders, including depression/anxiety disorders, schizophrenia, fear disorders (PTSD) and neurodevelopment disorders (ADHD and ASD).

### 3.1. Depression/Anxiety Disorders

Depression is one of the principal contributors to disability worldwide. This condition is present in all age groups and all socio-professional categories, with considerable morbidity and mortality, and affects one in seven people during their lifetime [67,68]. In psychiatric pathology, a characteristic depressive episode is referred to as a major depressive episode. The core features of depression are “depressed mood” and “loss of interest or pleasure in nearly all activities”, with symptoms of fatigue, sleep disturbance, anxiety, and neurocognitive and sexual dysfunction also worthy of monitoring [67]. Depression is also linked with numerous somatic and psychological comorbidities, such as smoking, chronic alcoholism and reduction in physical activity, which emphasizes its importance to public health. The main risk of depression is suicide. The risk of attempted suicide is increased in the event of a depressive episode. In depressed outpatients, a lack of improvement has been strongly associated with short-term attempts at suicide, particularly in patients with a history of previous attempted suicides [69,70].

Anxiety disorders, defined as the anticipation of future threat in the *Diagnostic and Statistical Manual of Mental Disorders* (DSM), *Fifth Edition* (DSM-V), comprise one of the major groups of disorders in psychiatry. Anxiety is distinguished from fear, the emotional response to real or perceived imminent threat [71]. Recent years have seen increased attention to providing a more sophisticated scientific approach to the possible etiology and pathology of anxiety disorders [72].

#### 3.1.1. Iron Deficiency-Related Depression/Anxiety Disorders

The causes of depression include biological, psychological and psychosocial or environmental factors [73]. Of note, lower levels of circulating iron have been reported to be associated with depression across different age groups and genders [74]. Fatigue, lethargy and depression can all be symptoms of iron deficiency [75]. One study examining 2000 persons over 65 inferred that lower levels of serum ferritin, transferrin and hemoglobin were linked with depressive symptoms [76]. Numerous studies have linked symptoms of depression with decreased levels of serum ferritin in adults, including Japanese workers [77,78], female medical students [79] and female soldiers [80]. In adolescents aged 12 to 17, serum ferritin was inversely correlated with depressive symptom severity [81]. Children with severe, chronic iron deficiency in infancy have increased anxiety and/or depression along with social and attention problems [82]. In addition, consistent results have been observed in the context of maternal IDA patients, where impaired postpartum reasoning and emotional reactions, and even postpartum depression, can occur [83,84]. The fact that treatment with iron supplements can improve the key aspects of mood in a combined cohort, including symptoms of depression, validates the described correlations between iron levels and psychological conditions [80,83,84]. It is important to note that limited studies revealed the relevance of brain iron deficiency and depression/anxiety disorders. For instance, in a prospective and observational study, it was found that individuals with depression had a significantly higher prevalence of hypotransferrinemia < 2 g/L, which may indicate a novel form of brain functional iron deficiency [24].

Animal studies have also revealed the relevance of iron deficiency to depressed mood or depression. Two separate studies found that a low-iron diet can result in depressed mood in monkeys [85,86]. Although these studies did not identify the specific molecular mechanisms involved, they did discuss the association between iron and the synthesis of monoamine transmitters, as discussed in Section 4.1 of this review. Rodent studies linked depression and iron management guidelines as well. In postpartum rats, gestational iron supplementation ameliorated symptoms of depression. The authors also demonstrated that iron supplementation prevented neuronal loss and increased dendritic spine density in the rat brain. The overall findings that perinatal iron supplementation could reduce susceptibility to postpartum depression, while iron deficiency had the opposite effect, are consistent with human studies [87].

Growing evidence suggests that iron plays an important role in the neurological mechanisms underlying anxiety disorders. Individuals with anxiety disorders tend to be more iron-deficient than healthy individuals [81]. A longitudinal follow-up analysis showed that children who had been tested and treated for iron deficiency as infants have greater anxiety/depression and social discomfort. These patients also presented lower scores for general mental and motor functions, as well as differences in some cognitive processes, compared with controls [82]. Another clinical study shed light on the close relationship between low iron availability and anxiety disorders. A significantly higher risk of anxiety disorder, delayed development and intellectual disabilities was noted among children (below age 13) with IDA. Adolescents with IDA aged 13 to 18 exhibited an increased risk of unipolar depressive disorder, anxiety disorder, delayed development, and other emotional illness. These results suggest that iron deficiency, whether in childhood or adolescence, does indeed have a significant impact on psychiatric comorbidities [88].

In rodent models, rats fed an iron deficient diet showed more anxiety-like behavior than control rats, which is consistent with observations reported in human studies [89]. Another study revealed that prenatal iron deficiency can also lead to elevated anxiety behavior in neonatal rats, which seemed to be rescued by iron repletion; however, some aspects of altered exploratory behavior and growth persisted into adulthood [90]. Despite the fact that effects of maternal iron deficiency in rats may differ from humans, given the differences in the timing of brain growth between the species, the study’s results may have implications for humans [91]. An additional study using *Cp*-deficient mice showed that the knockout (KO) mice exhibited a heightened anxiety phenotype, without any impairment of memory acquisition or retention [92]. The authors also considered whether the reduced hippocampal iron in Cp-deficient mice may result in reduced levels of serotonin, norepinephrine, and decreased brain-derived neurotrophic factor (BDNF) expression, which may underlie the sensitive anxiety behavior in Cp-deficient mice.

#### 3.1.2. Iron Overload-Related Depression/Anxiety Disorders

Compared with the relatively abundant research that links iron deficiency to depression, only a few studies have documented the influence of increased iron on depression and depressive-like behaviors. Treatment of a small group of psychiatric patients, who were found to have iron overload as manifested by abnormal ferritin, transferrin saturation index, or excessive urinary iron, with an iron chelator apparently led to clinical improvement [93]. A study in transfusion-dependent thalassemia children (TDT) aged 6 to 12 with iron overload showed that blood transfusions may be causally related to major depressive-like episodes in this population and that the effects are, at least in part, mediated by iron overload and the consequent immune-inflammatory response [94], this study is consistent with a previous report, which also reported the severity of depression in beta thalassemia patients [95]. In another study, researchers used quantitative susceptibility mapping (QSM) to examine brain iron concentration in depressed patients and evaluated whether iron was related to disease severity. The authors concluded that brain iron deposition may be associated with depression and may even be a biomarker for investigating the pathophysiological mechanism of depression [96]. Several similar findings are also found in late-life depressive patients on antidepressant medication and in patients with recurrent depression. It is reported that elevated iron concentration was found in several brain regions with the progress of depression [97,98].

In addition to the above clinical studies, an animal study in chronically stressed mice with depressive-like phenotypes demonstrated that iron overload exacerbated the depressive-like behaviors, and introduced motor and cognitive impairments that do not occur in mice exposed to chronic unpredictable mild stress (CUMS) alone [99,100]. Together, the above-mentioned studies indicate that increased iron uptake can exacerbate the pathophysiological evolution of depression and may suggest that excess iron uptake has to be considered as an additional risk factor for cognitive impairment in patients with depression.

There are few human studies focused on revealing the relevance of iron deposition and anxiety disorders. It is reported that children with thalassemia are also likely to have psychological problems like anxiety disorder or generalized anxiety disorder [101,102]. Furthermore, it has been observed that individuals with PD and anxiety exhibit increased iron accumulation in the fear circuit of the brain. This finding suggests that the excessive iron buildup in this region may potentially contribute to the onset and development of anxiety symptoms in individuals with PD [103]. Moreover, rodent studies have reported that iron overload in the brain appears to accelerate anxiety-like behavior and mood. Adult rats receiving daily intraperitoneal injections of iron [104,105] or being fed a carbonyl iron diet containing 20,000 ppm iron [106] both showed anxious responses and/or other behavioral impairments. After deletion of TfR2, which modulates systemic iron metabolism through the regulation of hepcidin, mice showed increased anxiety-like behaviors and brain iron availability. It is worth noting that these anxiety-related behaviors were iron dependent because they could be reverted by an iron deficient diet in Tfr2-KO mice [107]. An additional study reported that an axonal iron transport pathway from the ventral hippocampus (vHip) to the medial prefrontal cortex (mPFC) to the substantia nigra can modulate anxiety-related behaviors. Moreover, vHip-mPFC iron transport is essential for the anxiolytic effects of diazepam. These findings underscore the vital role of brain iron transport and identify it as a potential target for anxiety treatments [108]. Taken together, these findings support the hypothesis that imbalanced iron metabolism plays a pivotal role in modulating anxiety and emotional behaviors.

### 3.2. Schizophrenia

Schizophrenia is a severe mental disorder that can cause a combination of hallucinations, delusions, and severely disordered thinking and behavior that impairs daily functioning, and may be disabling. People with schizophrenia may perceive reality abnormally and often have persistent difficulties with cognitive skills such as memory, attention, and problem-solving. Worldwide, approximately 24 million people, or 1 in 300 people (0.32%), are affected by schizophrenia, and the rate is 1 in 222 people (0.45%) among adults. Unfortunately, stigma, discrimination, and violations of human rights in both mental health institutions and community settings against individuals with schizophrenia are common [109,110,111]. Research has not identified a single definitive cause of schizophrenia, but it is believed that an interaction between genes and environmental factors, including psychosocial factors, may contribute to the development of schizophrenia [112,113].

#### 3.2.1. Iron Deficiency-Related Schizophrenia

Iron is demonstrated to affect the pathophysiology of schizophrenia through several mechanisms, which will be further summarized in the fourth section of this review. Iron deficiency and schizophrenia have been linked in multiple studies, suggesting that there may be a potential relationship between the two conditions. A recent narrative review systemically reviewed the current state of science regarding perinatal iron deficiency and the development and pathophysiology of schizophrenia. The authors proposed that perinatal iron deficiency may act as a potential early environmental insult in the developmental programming of schizophrenia, with evidence from animal, human, and epidemiological research [114]. A meta-analysis of 39 studies combined plasma and serum data, providing evidence of deficient iron in patients with schizophrenia [115]. Additionally, decreased iron concentration was found in gray matter nuclei in patients with first-episode schizophrenia, using QSM and effective transverse relaxation rate (R2*) maps [116]. Furthermore, iron supplementation has been shown to improve cognitive function and symptoms in patients with schizophrenia who were also iron deficient [117]. These findings suggests that there may be a potential clinical use for iron supplementation in the treatment of schizophrenia.

#### 3.2.2. Iron Overload-Related Schizophrenia

Recent studies have suggested a potential relationship between iron overload and schizophrenia symptoms. Magnetic resonance brain neuroimaging research in schizophrenia patients has shown elevated brain iron levels in different brain regions, including the putamen and thalamus [118,119]. In addition, increased iron levels were also found in the prefrontal cortex of people with schizophrenia, as measured by inductively coupled plasma-mass spectrometry (ICP-MS) and Western Blots [60]. This emerging evidence suggests a potential association between iron overload and schizophrenia in individuals or in post-mortem human brain samples. However, the exact mechanisms through which iron overload or deficiency contributes to schizophrenia are not yet fully understood. It is believed that iron accumulation in certain brain regions may disrupt neurotransmitter systems or myelination involved in cognition and emotion regulation [120]. Additionally, high levels of iron may contribute to oxidative stress and inflammation, which have been implicated in the pathophysiology of schizophrenia [121,122]. 

Further research is needed to understand the complex relationship between iron metabolism and schizophrenia. Identifying and treating iron overload or deficiency in these cases may help alleviate psychiatric manifestations, but more research is needed to elucidate the underlying mechanisms and explore potential therapeutic interventions.

### 3.3. Post-Traumatic Stress Disorder (PTSD)

Fear memory is universally present in humans and several animal groups. This memory is a self-protective reaction that evolved to enable adaptation to the environment, and, ultimately, survival. Although fear has a strong survival value, excessive fear stress response can surpass the needs of the individual to effectively avoid danger and acquire coping strategies, leading to the occurrence of pathological mental disorders, such as PTSD [123,124]. PTSD is a disabling neuropsychiatric disorder characterized by the intrusion of an event, avoidance of recall of an event, alterations in cognition and mood, and hyperarousal, as per the DSM-V. In PTSD, fear memory becomes recurring and may be recalled very intensely in the form of flashbacks in circumstances in which the sufferer recognizes elements or components of the traumatic experience, even in the absence of stimuli that resemble the conditional stimuli. Therefore, PTSD is viewed as a serious psychiatric condition in which the most prominent symptom is a widely generalized retrieval of fearsome and traumatic experiences [123].

#### 3.3.1. Iron Deficiency in the Pathology of PTSD

Brain iron plays a vital role in the function of learning and memory. An increasing number of studies have found that aberrant iron metabolism is involved in PTSD pathology. A follow up in adolescents from a study in infants reported that iron deficiency at 12 or 18 months of age predicted greater adolescent behavioral issues compared with iron sufficiency; the iron deficient infants exhibited increases in adolescent-reported anxiety and social problems, and parent-reported social, PTSD and attention-deficit/hyperactivity disorder (ADHD) [125]. A study employing a trace heart rate-fear conditioning paradigm demonstrated that perinatal nutritional iron deficiency impaired hippocampus-dependent fear learning, which was not reversed by a subsequent normal iron diet, and appeared to persist into adulthood [126]. Investigation of gestational iron deficient rats revealed diminished fear conditioning in postnatal (P) rats on days P28 and P35. Serendipitously, this research also found that trace fear conditioning was enhanced by prior iron deficiency in the P56 group. This unanticipated finding in iron-repleted adults is consistent with the effects of developmental iron deficiency on inhibitory avoidance learning, but contrasts with the persistent deleterious long-term effects of a more severe iron deficiency protocol, suggesting that the degree and duration of iron deficiency can affect the possibility of recovery from its deleterious effects [127]. In our recent study, we investigated the affected contextual fear memory caused by brain iron deficiency in genetically modified mice. After specific deletion of FPN1 in neurons and astrocytes, the total iron contents in the cortex and hippocampus were dramatically decreased. The deficient brain iron, which was subsequently shown to be a consequence of decreased iron delivery through the BBB, ultimately depressed the contextual fear memory. The study highlights the major role played by FPN1 in brain iron homeostasis and identifies this pathway as a potential target for the treatment of PTSD [26].

#### 3.3.2. Iron Overload in the Pathology of PTSD and Fear Memory

Iron overload may also function in fear memory and in the pathology of PTSD. In peripheral tissues, chronic psychological stress in mice led to significantly increased iron content in plasma and the duodenum, whereas the hepatic iron level was markedly decreased, providing insights into the mechanisms of iron metabolism during the stress disorder [128]. In a rat model of PTSD, stress exposure increased the level of total brain iron, and TfR1 and ferritin expression in the hippocampus, frontal cortex, and striatum, all of which are associated with fear memory treatment. The elevated iron in these cognition-related brain regions may be responsible for the neuronal injury, suggesting that iron may function in the pathology of PTSD [129]. In addition, the loss of FPN1 in neurons, which leads to brain iron accumulation, impairs fear memory by promoting ferroptosis in Alzheimer’s disease (AD) [130]. It is worthy to point out that, in a neonatal iron overload model in rats, permanently elevated brain iron also impaired inhibitory avoidance memory, an instrumental fear conditioning memory [131,132,133,134]. In another study, anesthesia-induced brain iron overload or iron deficiency also led to fear memory deficits with different mechanisms that warrant further elucidation [135,136].

### 3.4. Neurodevelopment Disorders

#### 3.4.1. Brain Iron and ADHD

ADHD is a neurodevelopmental disorder characterized by excessive amounts of inattention, carelessness, hyperactivity (which can evolve into inner restlessness in adulthood), and impulsivity that are pervasive, debilitating and otherwise age-inappropriate [137]. Some individuals with ADHD also display difficulty regulating emotions and have executive dysfunction. ADHD is also associated with other mental disorders and substance abuse disorders, which can cause additional impairment [138,139].

Limited research has been conducted on the impact of brain iron overload on ADHD. Nevertheless, several studies have established a correlation between iron deficiency (ID), particularly serum iron deficiency, and ADHD [19,140,141]. To establish a potential correlation between iron deficiency in specific brain regions and ADHD symptoms, larger, longitudinal magnetic resonance imaging (MRI) studies were also conducted, offering a deeper understanding of the underlying mechanisms. In a pilot MRI study, children aged 8–14 years with ADHD exhibited significantly lower estimated brain iron levels in both the right and left thalamus compared to healthy controls. Furthermore, these children also demonstrated significantly lower levels of serum ferritin compared to the control group [142]. Similar findings have been reported in different brain regions, including the thalamus, using non-invasive MRI methods [143]. Another whole-brain analysis using QSM revealed iron deficiency in several brain regions in children with ADHD, such as bilateral striatums, anterior cingulum, olfactory gyrus, and right lingual gyri [140]. These studies suggest that iron deficiency may play a role in the pathophysiology of ADHD.

The long-term association found between infant iron deficiency and sluggish cognitive tempo and attention-deficit/hyperactive-impulsive behaviors suggest that the neurodevelopmental alterations that stem from postnatal iron deficiency may play an etiological role in the development of ADHD [144,145]. A recent, systematic review including a meta-analysis of three studies, in which brain iron content was assessed, revealed a statistically significant, lower thalamic iron concentration in children with ADHD than in healthy control subjects [146]. A double-blind, randomized, placebo-controlled trial investigating the effects of oral ferrous sulfate on ADHD revealed significant improvement in subjects who received iron supplementation therapy. The improvements were observed in the total score as well as the hyperactive/impulsive and inattentive subscales of the ADHD Rating Scale, a standardized assessment tool used to evaluate ADHD symptoms [147]. These findings suggest that iron supplementation (at a dosage of 80 mg/day) may be beneficial for children with low serum ferritin levels, indicating the need for further investigation through larger controlled trials. Importantly, iron therapy was well-tolerated and demonstrated comparable effectiveness to stimulant medications [147]. Additionally, a qualitative systematic review of nine randomized clinical trials examined the efficacy of iron-zinc supplementation in the treatment of ADHD in children and adolescents [148]. The review findings indicated that lower baseline levels of zinc and iron were associated with higher ADHD severity and poorer treatment outcomes. Dietary supplementation with zinc and iron showed improvements in ADHD symptom severity compared to the placebo control, although the effect sizes were small and specific to certain ADHD symptoms. This suggests that iron–zinc supplementation may be beneficial for specific subgroups of individuals with ADHD [148]. Taken together, these findings further support a potential association between reduced brain iron concentration and ADHD in this specific age group and highlight the importance of early intervention and treatment to prevent or minimize the impact of this condition on an individual’s cognitive and behavioral functioning. However, further research is required to confirm and expand upon these results.

#### 3.4.2. Brain Iron and ASD

ASD is a range of neurodevelopmental conditions primarily characterized by significant difficulties in social interactions, differences in communication, and rigid and repetitive behavior. Unusual responses to sensory input, including high or low sensitivity, sensory discrimination, and sensory-based motor differences, are also highly prevalent [149].

Previous studies have raised concerns that the intake of the trace elements, iron and zinc, vitamins (B vitamins, vitamin D, vitamin K), and calcium may be deficient in up to one-third of children with ASD [150,151]; children with ASD are at risk for low serum ferritin, which has been associated with attention and sleep issues [152,153]. As with the other neuropsychiatric disorders discussed herein, the association between brain iron status and ASD is also unclear, i.e., whether a lower-than-normal level of serum iron may result in a lower-than-normal level of brain iron requires confirmation. A study measuring brain iron contents in various regions using QSM showed lower brain iron content in preschool children with autism than normal preschool children [154]. A population-based case–control study revealed that children with autism exhibited neurodevelopmental delays, and provided initial evidence for an association between increased maternal supplemental iron intake and reduced risk of ASD [155]. Also, abnormalities in brain iron may contribute to various psychiatric disorders, including autism with altered myelin-related molecular systems [62,153]. Based on the previous findings, Table 1 presents an overview of the current progress in clinical evidence regarding the relationship between brain iron (Fe) and the aforementioned mental disorders.

## 4. Potential Mechanisms Underlying the Influence of Iron on Mental Disorders

**Figure 2:**
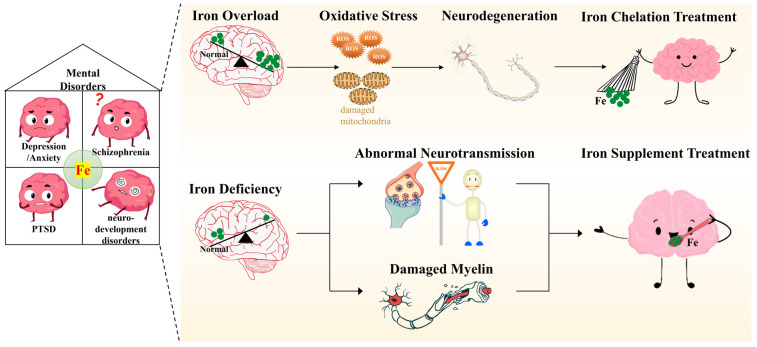
Potential mechanisms underlying the pathological processes of mental health conditions and targeting brain iron metabolism in the treatment of related mental disorders.

Numerous studies, from animal models to humans, have considered the effects of peripheral and central iron status on mental disorders. However, limited research has focused on revealing the potential molecular mechanisms of iron’s involvement in the pathogenesis or pathology of such diseases. Iron’s role in physiological functions includes, but is not limited to, energy metabolism, neurotransmission and myelination. These functions also have important roles in the development of mental disorders. For instance, altered dopaminergic and serotonergic neurotransmission have been described in depression, anxiety-related disorders, ADHD and ASD [156,157,158]. An accumulating body of evidence also implicates both energy and myelination, both of which are affected by iron status, in mental disorders [159]. While there may be additional mechanisms linking iron to brain function, since there is a relatively clear relationship between iron status and neurotransmission, oxidative stress and myelination, we will focus on these three mechanisms by exploring the literature linking iron to mental disorders (Figure 2).

Imbalanced brain iron metabolism, including iron overload and iron deficiency, is involved in the pathological processes of mental disorders, including depression, anxiety, schizophrenia, PTSD and neurodevelopment disorders. Brain iron overload, seen in depression, anxiety, schizophrenia and PTSD, results in elevated oxidative stress that can damage mitochondria, ultimately leading to neurodegeneration. Sequestering excess brain iron using exogenous iron chelators is becoming a viable strategy in the treatment of iron overload-related mental disorders, such as cognitive impairments, depression and anxiety. Moreover, brain iron deficiency is particularly relevant to the pathogenesis of neurodevelopment disorders, including ADHD and ASD, depression, anxiety, schizophrenia and PTSD. Insufficient brain iron results in abnormal neurotransmission and neurodevelopment due to both a lack of neurotransmitters and damaged myelin. Accordingly, iron supplementation may also be a successful approach in the clinic.

### 4.1. Iron and Neurotransmission

Mounting evidence supports an intimate connection between brain iron status, especially iron deficiency, and dysfunction in both myelination and neurotransmission, especially monoamine metabolism [160]. Monoamines, including serotonin, dopamine (DA) and noradrenaline (NA), have major roles in various aspects of emotional behaviors. Indeed, the balance of these three neurotransmitters (NTs) can determine various mental disorders [76,161].

Several studies have focused on how brain iron status affects the neurotransmission mediated by monoamines. Iron plays a pivotal role in the synthesis of serotonin, DA and NA, as the metal is an essential cofactor of aromatic amino acid hydroxylases (phenylalanine, tyrosine, tryptophan [Trp]). These enzymes can limit the synthesis of the above-mentioned monoamines. Neuronal iron deficiency decreased the availability of Trp in neurons, resulting in lower synaptic serotonin concentration. Dysregulation and diminished serotonin concentration then reduced the synaptic connectivity in the prefrontal cortex and hippocampus, ultimately triggering neuro-behavioral manifestations, neuropsychological disorders and depression [24]. A study in humans revealed that children and young adults with IDA in infancy show altered social-emotional behavior, specifically wariness and hesitance, a lack of positive affect, and diminished social engagement, among other behavioral abnormalities. Pharmacologic and neuroimaging studies implicate an altered mesolimbic dopamine pathway [162]. Studies employing iron-deficient rodent models have also demonstrated a robust relationship between behavioral abnormalities and altered dopamine metabolism in the brain [163,164]. It has also been documented that iron metabolism is involved in the dopaminergic pathway and neurotransmission. DA anabolism involves the iron-dependent phenylalanine and tyrosine hydroxylases (TyrH). Reduced activity of TyrH caused by iron deficiency, especially in the substantia nigra, can impair DA synthesis, which may then cause psychological and motor issues in adults [165,166,167].

Compared to the various studies on the consequences to DA affected by brain iron metabolism, few studies have examined serotonin and NA signaling. However, alterations in serotonin signaling may also be responsible for emotional behaviors since the neurotransmitter has an important role in mediating affective behaviors. A study in patients with Parkinson’s disease (PD) revealed an early, linear association between low serotonin and higher nigral iron, which was absent in controls, warranting further studies to investigate the relationship between brain iron status and serotonin-related emotional behaviors in the pathology of PD [168]. In animal studies, differing results have been observed. Brain iron deficiency can both up-regulate or decrease the levels of serotonin in different rat models [169,170]. Additionally, in other rat studies, diminished brain iron levels had no effect on serotonin levels or metabolism in newborns or adults, and brain serotonin in the iron-deficient rats did not differ from controls [171,172]. Extracellular NA concentrations are elevated under iron deficiency states, whereas tissue levels are unchanged compared with controls [173,174,175]. A diverse set of results from NA transporters (NAT) and α2 adrenergic receptor expression was observed in ID rats [176]. In CP deficient mice, increased anxiety is associated with elevated levels of plasma corticosterone and decreased levels of serotonin and NA, as well as brain-derived neurotrophic factor (BDNF) and its receptor [92]. Altered hippocampal BDNF signaling is linked to changes in serotonin and NA levels. Reduced BDNF and increased corticosterone are associated with increased anxiety [177,178]. These observations of monoamine homeostasis affected by brain iron status are not necessarily conflicting, but may hint that there is particular significance to the molecular mechanisms around the iron–monoamine relationship in emotional behaviors.

### 4.2. Iron and Oxidative Stress

Iron plays important roles in cell biology, including oxygen transport, storage, and delivery in the form of heme in hemoglobin and myoglobin. The heme cofactor is also a major component in numerous oxidase and oxygenase enzymes, and in electron transfer proteins (cytochromes) for mitochondrial electron transport [179,180]. Due to iron’s unique redox properties, coupled with the high-energy demand of the brain, brain iron must be strictly regulated. Iron can induce oxidative stress by catalyzing the formation of hydroxyl radicals, the most harmful cellular reactive oxygen species (ROS), through the Fenton reaction [181]. ROS can directly damage DNA, cause several direct oxidative modifications of amino acid residues, and promote the peroxidation of polyunsaturated fatty acids in membrane lipids, leading to alterations in and functional loss of membranes [16,182].

TDT patients with iron overload exhibit severe depression, which may be related to increased iron-induced oxidative stress and immune inflammation [94,183]. In a rat model of PTSD, increased iron levels in the hippocampus, frontal cortex and striatum were shown to lead to mitochondrial swelling and neuronal cell apoptosis through increased oxidative stress [129]. Another mouse study demonstrated that hippocampal iron overload, induced by the deletion of the iron trafficking protein lipocalin-2 (LCN2), led to elevated oxidative stress, thus interfering with neurogenesis in adult animals and ultimately abolishing hippocampal-dependent contextual fear discriminative task behavior. Of note, supplementation with the iron-chelating agent, deferoxamine, rescues oxidative stress in adult neural stem cells (NSCs), promotes cell cycle progression and improves contextual fear conditioning [184]. Additional studies have reached similar conclusions that brain iron overload may cause mood disorders through oxidative stress [185,186].

Excess iron-induced oxidative stress can also have an impact on monoamine function. Administration of a small amount of iron for only a few days promoted iron accumulation in the substantia nigra of the adult brain, where iron may generate cytotoxic free radicals. Such oxidative stress can impair dopaminergic signaling and monoamine function, leading to behavioral impairments [186,187,188]. M30, an inhibitor of monoamine oxidase A, can attenuate chronic intermittent hypoxia-induced oxidative stress, inflammation and serotonin deficiency by chelating excess iron, and may prevent the onset of depressive symptoms in obstructive sleep apnea patients [189]. In addition, ferroptosis, a newly recognized type of cell death caused by the accumulation of intracellular iron that promotes lipid peroxidation and then cell death, may also have an influence on mental health and related emotional disorders [130,190,191]. Iron-related oxidative stress also participates in the pathology of neurodegenerative diseases and the related cognitive dysfunction, such as in AD and PD [192]; however, the exact role of iron and oxidative stress in emotional disorders require further clarification.

### 4.3. Iron and Myelination

Various studies have reported perturbations of myelin and myelin-related systems in psychiatric disorders, with altered myelination during childhood and adolescence implicated in schizophrenia, autism and other conditions [193,194]. Altered myelination can affect synapse formation and its plasticity, conduction velocity and synchronic impulse trafficking between different brain regions. This may both influence normal mental performance and contribute to psychiatric symptoms in disorders involving myelin abnormalities [195]. As iron is directly required for myelination, it has been shown that poor brain myelination resulting from iron deficiency in early development has long-lasting effects on behavioral functions [196,197,198]. A few studies have examined the relationship between emotional disorders and interdependent abnormalities in brain iron metabolism and myelin, or the molecular systems involved. A study using a mouse model of increased brain iron levels demonstrated that brain iron accumulation affects myelin-related molecular systems implicated in neurodegeneration with brain iron accumulation (NBIA) with neuropsychiatric features [62]. These studies are indicative of a link between brain iron status and myelination, whose detailed mechanisms still require in-depth understanding.

## 5. Limitations of the Presented Studies

While the above review provides important insights into the association between iron deficiency and iron overload with mental disorders, we must also take note of the limitations of these studies. Firstly, most of the studies are based on observational data and cannot establish causality. Although there are some experimental studies, more randomized controlled trials are still needed to validate these findings. Secondly, different methods of behavioral measurements and the influence of other metals may also produce different behavioral outcomes [161]. There are variations in the psychological health assessment tools and iron indicators used in the studies, limiting the comparability of results. Thirdly, there are differences in the characteristics of the study samples, such as age, gender, and baseline iron levels, which may affect the interpretation and generalizability of the results. Additionally, although animal studies provide valuable information for exploring the relationship between iron and mental disorders, there are biological and physiological differences between humans and animals, so these findings need to be cautiously interpreted and extrapolated. Moreover, studies exploring the relationship between mental disorders and changes of endogenous brain iron metabolism are rare. Therefore, further research on patients with gene mutation that can affect the brain iron metabolism should be carried out to reveal its relevance. Fourthly, the occurrence and development of mental disorders is a complex process involving the interaction of multiple factors, such as genetics, environment, and lifestyle. Iron is just one factor, and other factors, like other trace elements, may also play important roles in the occurrence of mental disorders. Finally, the use of iron supplementation and iron chelation therapy holds great promise in the treatment of psychological disorders, but it also has its limitations. The action of medications often affects a wide range of areas in the body, while iron deficiency or iron overload typically occurs in specific regions of the brain in the pathophysiology of mental disorders. However, our research has found that liposome-based DFO (Deferoxamine) has shown promising results in the treatment of cerebral ischemia, which suggests that liposome-based iron chelators with their targeted nature provide a valuable avenue for research [199]. Therefore, the development of targeted therapeutic agents for the treatment of psychological disorders is a highly promising and necessary research direction. Further research needs to employ more rigorous study designs, control for potential confounding factors, and conduct larger-scale, multicenter studies to better understand the relationship between iron and mental disorders and provide more reliable evidence for prevention and treatment. We must improve our understanding of the pathologies associated with other psychiatric disorders as well to develop therapies to alleviate mental dysfunction.

## 6. Conclusions and Prospects

As a group, mental disorders have a generally high incidence, compared with other health conditions. According to the World Health Organization (WHO), 970 million people around the world live with a mental disorder, with anxiety and depressive disorders being the most common [200]. In 2020, the number of people living with anxiety and depressive disorders rose significantly as a result of the COVID-19 pandemic [64]. While effective prevention and treatment options exist, most people with mental disorders do not have access to effective care. Untreated mental health conditions can lead to severe personal consequences, such as self-harm and suicide, creating significant familial and societal burdens [201,202]. Individuals with mental disorders can also experience stigma, discrimination and a violation of human rights [203,204,205]. In view of this, uncovering effective prevention and treatment options has become a major aim in a multitude of research programs.

Iron has specific and unique roles in the brain because of the metal’s direct involvement in the CNS function, including myelin synthesis, neurotransmitter synthesis and metabolism [59]. Imbalanced brain iron metabolism, such as iron overload, can participate in and even aggravate the pathology of neurological diseases, such as AD, PD and stroke, through elevating oxidative stress that can destroy the cell membrane and induce cell death [16,206,207]. On the other hand, iron deficiency and brain iron deficiency are correlated with mental disorders through impairing neurodevelopment, largely in pathways related to neurotransmission and myelination. Moreover, in the treatment of diseases induced by imbalanced brain iron homeostasis, numerous studies have suggested that exogenous modulation of iron metabolism may be successful at preventing or treating neurological disorders. For instance, iron chelators have been reported to alleviate the symptoms of AD, PD and stroke, or show therapeutic promise in preclinical and clinical models of other neurological disorders [208,209,210]. In contrast, iron supplement has been suggested to relieve ID-related mental disorders [211,212]. Finally, several key molecules of brain iron metabolism, such as hepcidin and CP, have also been reported as potential therapeutic targets for AD and PD through their neuroprotective roles in the pathological processes of these diseases [16]. Expanding our understanding of brain iron metabolism is crucial to affecting underlying mechanisms in order to improve the strategies that help prevent and possibly cure mental disorders.

The mechanisms underlying the influence of iron on mental disorders are various and complicated. The effects of iron in psychological health are determined by numerous physiological/biological properties and spatial/temporal factors. These include neurotransmission, iron-related myelination and oxidative stress, among many others. Recently, the influence of ferroptosis on mental health and related emotional disorders as also been illustrated in several studies [130,190,191]. In addition, iron distribution in different brain regions, and the duration and timing of iron exposure (i.e., during different development periods), the route of exposure, animal species, sex, nutritional status and disease state all can contribute to the influence of iron on mental disorders.

Previous studies on iron homeostasis and mental health conditions revealed that targeting brain iron metabolism-related molecules, particularly those relating to iron supplementation, for drug development is expected to identify innovative ways to treat neuropsychological disorders [80,83]. In a previous, recent work, we reviewed the methods of iron supplementation and iron chelation, as well as the latest progresses in this area [16]. With the continuous developments in brain iron metabolism and mental disorders, the relationship between iron and the brain is becoming a rich area for translatable scientific exploration. Important connections between mental health conditions and other trace elements, such as copper and zinc, are also becoming apparent [213,214]. It is therefore expected that a novel discipline of trace element nutrition and mental disorders will emerge as a new direction for clinical therapy.

## Figures and Tables

**Table 1:** Summary of current clinical evidence for the relationship between brain iron (Fe) and mental disorders.

Mental Disorders	Participants/Samples	Brain Iron Levels	Methods/Strategies	Key Findings	References
Depression	Depressed patients	Elevated (putamen, thalamus)	quantitative susceptibility mapping (QSM)	The study indicates the role of excess brain iron in deep gray matter in depression. It also suggests iron may be a potential biomarker for further understanding the pathophysiological mechanism of depression.	[96]
Depression	Late-life depressive patients (on antidepressant medication)	Elevated with the progression of depression: medial prefrontal cortex (mPFC), dorsal anterior cingulate cortex (dACC), occipital areas, habenula, brainstem, and cerebellum.	magnetic resonance imaging (MRI)-based QSM	It strengthens the understanding of the progression of brain iron deposition in late-life depression in patients on antidepressant medication and highlight the close relationship between magnetic susceptibility in the medial frontal areas and depression.	[97]
Depression	Patients With Recurrent Depression	Elevated (frontal lobes, temporal lobe structures, occipital lobes hippocampal regions, putamen, thalamus, cingulum, and cerebellum)	QSM	Brain iron deposition has been found to be associated with the overall duration of disease onset, rather than the severity of depression.	[98]
Depression	Depressive population	Decreased (hypotransferrinemia)	Blood iron detection	The hypotransferrinemia observed in the depressive population could correspond to a new form of brain functional iron deficiency.	[24]
Anxiety disorders	PD patients with anxiety	Elevated (ventral mPFC, ventral ACC, precuneus, angular gyrus, middle occipital gyrus, and supplementary motor area (SMA), hippocampus, and substantia nigra)	QSM	Increased iron accumulation in the fear circuit in PD patients with anxiety might contribute to the development of anxiety in PD.	[103]
ADHD	ADHD children aged 8–14 years	Decreased estimated brain iron levels in both the right and left thalamus	MRI	Low iron in the thalamus may contribute to ADHD pathophysiology	[142,143]
ADHD	ADHD children aged 6–14 years	Deficient iron in bilateral striatums, anterior cingulum, olfactory gyrus, and right lingual gyri	QSM	Brain iron deficiency in these brain regions might be related with ADHD, which might be valuable for further studies.	[140]
ASD	children with autism aged 2–3, 3–4, 4–5, and 5–6 years	Decreased iron contents (in caudate nucleus, dentate nucleus, and splenium of the corpus callosum for the 2–3 years group; in the frontal white matter, caudate nucleus, red nucleus, substantia nigra, dentate nucleus, and splenium of the corpus callosum for the 3–4, 4–5, and 5–6 years groups)	MRI enhanced T2*-weighted angiography (ESWAN) sequence scans	The brain iron content of children with autism is lower than that of normal children	[154]
Schizophrenia	Patients with first-episode schizophrenia	Decreased iron levels in the bilateral substantia nigra, left red nucleus and left thalamus	QSM effective transverse relaxation rate (R2*) maps	Decreased iron concentration is found in grey matter nuclei of patients with first-episode schizophrenia	[116]
Schizophrenia	an adult cohort of individuals with chronic schizophrenia aged 18–65 years	Elevated brain iron (thalamus)	inverse-normalized T2*-weighted contrast (1/nT2*)	Thalamic iron accumulation may act as a potential marker of schizophrenia	[118]
Schizophrenia	Post-mortem human brain samples	Elevated brain iron (the prefrontal cortex)	inductively coupled plasma-mass spectrometry (ICP-MS) Western Blots	It provides a pathophysiologic link between perturbed cortical iron biology and schizophrenia and indicates that achievement of optimal cortical iron homeostasis could offer a new therapeutic target	[60]
Schizophrenia	individuals with chronic schizophrenia	Increased iron in the putamen	ultra-high field 7 T QSM magnetic resonance spectroscopy (MRS)	Elevated iron levels in the dorsal striatum may be associated with a network-wide impact on iron distribution within other brain regions.	[119]

## Data Availability

All data is contained within the article.
